# A Case Report on Pericardial Effusion Associated With Selpercatinib in the First Patient With Rearranged During Transfection (RET) Fusion-Positive Lung Cancer

**DOI:** 10.7759/cureus.86275

**Published:** 2025-06-18

**Authors:** Tomoko Shiraishi, Yu Isoshima, Masahiro Tahara, Takanobu Jotatsu, Kazuhiro Yatera

**Affiliations:** 1 Department of Respiratory Medicine, University of Occupational and Environmental Health, Fukuoka, JPN

**Keywords:** adverse event, pericardial effusion, rearranged during transfection (ret) fusion-positive lung cancer, ret kinase inhibitor, selpercatinib

## Abstract

Selpercatinib was approved in 2021 for the treatment of rearranged during transfection (RET) fusion-positive lung cancer. The most frequent adverse events associated with selpercatinib are hepatic dysfunction, hypersensitivity, hypertension, QT interval prolongation, and peripheral edema. However, pericardial effusion has not been reported as an adverse event in the package insert or background clinical studies. A 70-year-old woman with RET fusion gene-positive lung cancer cT2bN2M1c (OSS, BRA) stage IVB developed pericardial effusion about 18 months after starting selpercatinib treatment. The patient wished to continue selpercatinib treatment, so the dose was gradually and carefully reduced to 40 mg/day. This dose reduction resulted in a decrease in pericardial effusion while maintaining tumor regression.

RET fusion-positive lung cancer is rare, accounting for only 1%-2% of all lung cancers. Since selpercatinib was approved relatively recently in 2021, the long-term adverse effects of its administration remain unclear. Long-term treatment with selpercatinib may potentially be associated with fluid accumulation, including pericardial effusion, although further studies are needed to clarify this relationship.

## Introduction

The rearranged during transfection (RET) gene is a proto-oncogene located on the long arm of chromosome 10 that was discovered in 1985 [1]. The transmembraneRET receptor-type tyrosine kinase encoded by the RET gene plays an important role in the development of the kidney and enteric nervous system during embryogenesis, and mutations in the RET receptor-type tyrosine kinase are involved in cancer cell growth [2].RET gene fusions, such as KIF5B-RET and CCDC6-RET*, *accounting for about 80% of RET fusion-positive non-small cell lung cancers (NSCLC), were first identified in lung cancer in 2012 [3-7]. RET fusion gene-positive lung cancer is relatively rare, accounting for only about 1%-2% of lung adenocarcinomas [4], and selective inhibitors of the RET-kinase have proven effective [8].

Selpercatinib was approved as a treatment forRET fusion-positive lung cancer by the United States Food and Drug Administration (FDA) in May 2020, and in Japan in September 2021. In the randomized LIBRETTO-431 study [9], it significantly prolonged progression-free survival (PFS) compared to platinum-based therapy or platinum-based therapy combined with pembrolizumab (24.8 vs. 11.1 months). In the registrational phase I/II, single-arm, open-label trial LIBRETTO-001, selpercatinib also demonstrated durable efficacy, with a median PFS of 26.2 months in previously treated patients and 22.0 months in treatment-naïve patients [10]. The most frequent adverse events of selpercatinib are hepatic dysfunction, hypersensitivity, hypertension, QT interval prolongation, and peripheral edema [10]. However, pericardial effusion has not been reported as an adverse event of selpercatinib, and its pathogenesis remains unclear [9-13].

Although selpercatinib offers relatively long-lasting clinical benefit, the rarity of RET fusion-positive NSCLC limits our current understanding of its long-term safety profile. Here, we report the first case report of a lung cancer patient with pericardial effusion developing after approximately 18 months of treatment with selpercatinib.

## Case presentation

**Figure 1:**
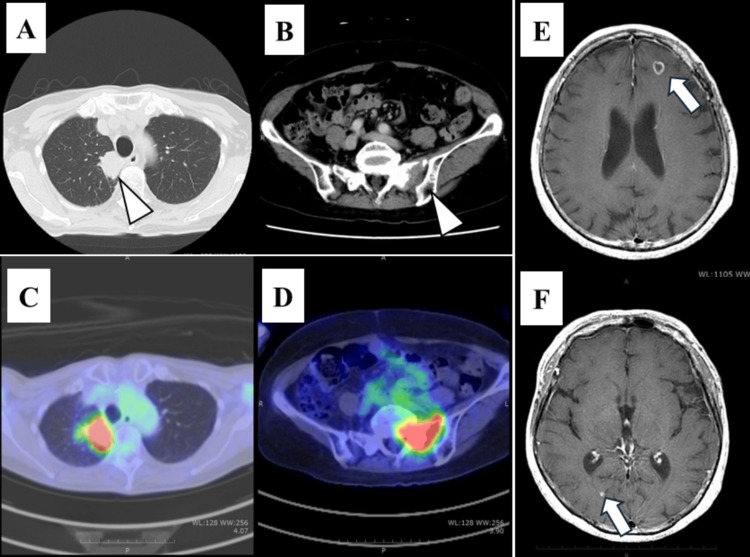
Radiological findings of the tumor at the initial examination (A) CT findings. A mass is seen in the upper lobe of the right lung (white arrowheads). (B) CT findings. Osteolytic changes associated with bone metastasis were observed in the sacrum near the sacroiliac joint. (C) ^18^F-FDG-PET/CT image showing that fluorodeoxyglucose uptake was increased in the mass in the upper lobe of the right lung. (D) ^18^F-FDG-PET/CT image showing that fluorodeoxyglucose uptake was increased in the sacroiliac joint area of the sacrum. (E,F) Contrast-enhanced magnetic resonance imaging of the brain shows enhanced nodules in the left subfrontal cortex and right occipital lobe (white arrows) ^18^F-FDG-PET/CT: ^18^F-2-fluoro-2-deoxy-D-glucose positron-emission tomography/computed tomography

A 79-year-old Japanese woman with a medical history of bronchial asthma, papillary thyroid cancer (total thyroidectomy was performed in 2012), and meningioma surgery had been followed due to postoperative secondary hypothyroidism, and thyroid hormone levels have remained normal with thyroid hormone replacement. Her body weight was 46.8 kg, and her height was 151 cm. A chest computed tomography (CT) revealed a mass lesion of the lung during the postoperative follow-up, and close examination of the lung lesion was initiated in October 2021. Her chest CT scan revealed a 48-mm-sized mass on the mediastinal side of the right upper lobe of the lung (Figure 1).

**Figure 2:**
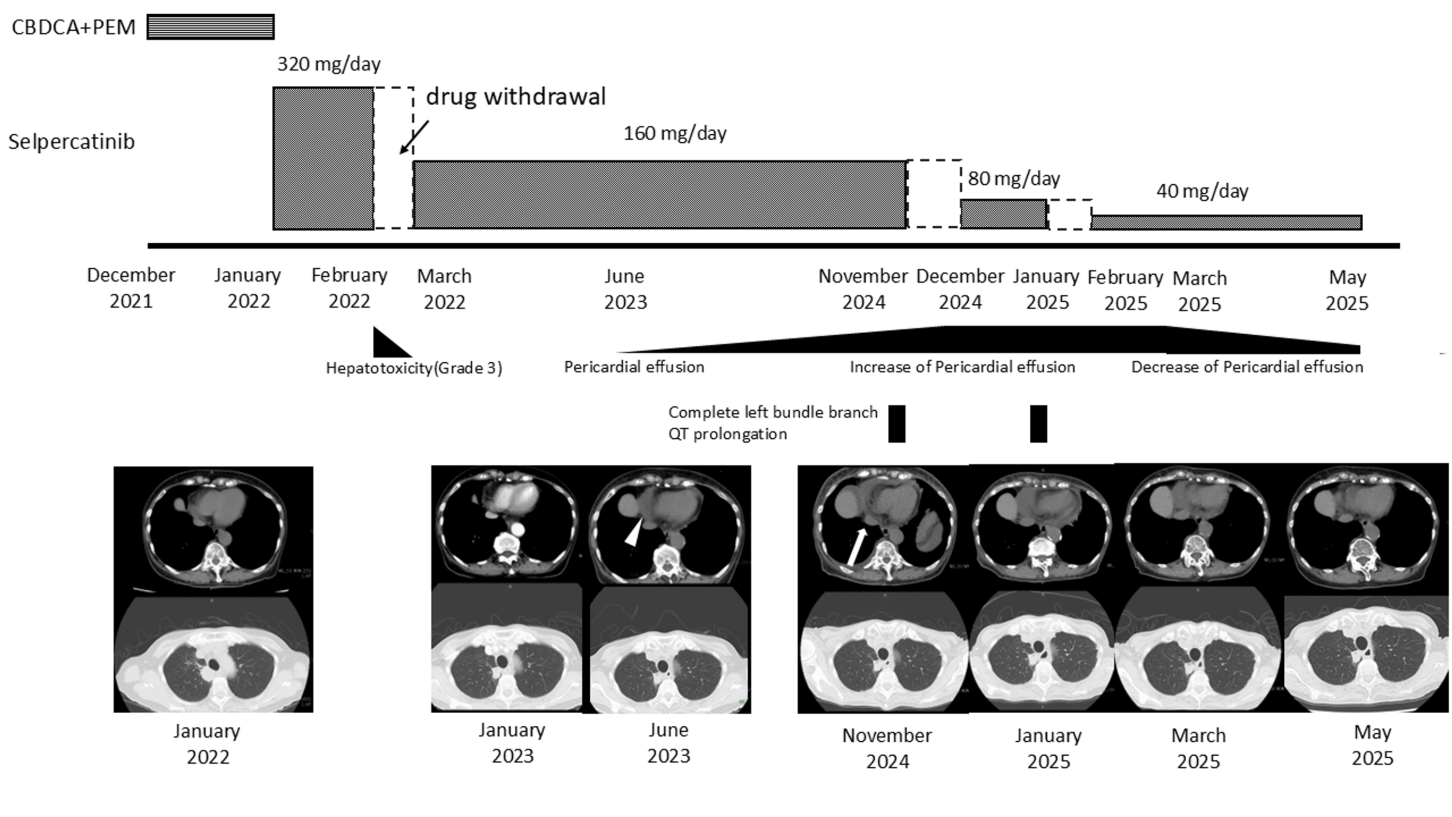
Clinical course CT image of June 2023 showed a small amount of pericardial effusion (white arrowheads). The CT image of November 2024 showed increased pericardial effusion (white arrow) and QT prolongation with complete left bundle branch block. After selpercatinib was reduced to 40 mg/day, complete left bundle branch block resolved, pericardial effusion decreased, and the tumor stabilized without progression CT: computed tomography

After a comprehensive evaluation, including a transbronchial lung biopsy, she was diagnosed with RET fusion gene-positive lung cancer, stage IVB (cT2bN2M1c with bone and brain metastases). Transthoracic echocardiography was performed before chemotherapy and showed no other abnormal findings, including pericardial effusion. Although selpercatinib was considered effective as a first-line treatment for her, it was not approved for use at the hospital at that time. Therefore, first-line treatment with carboplatin (CBDCA) and pemetrexed (PEM) was initiated in December 2021. Subsequently, after selpercatinib was approved at the hospital, the treatment was switched to selpercatinib (320 mg/day) in January 2022. The clinical course of our patient is shown in Figure 2.

**Figure 3:**
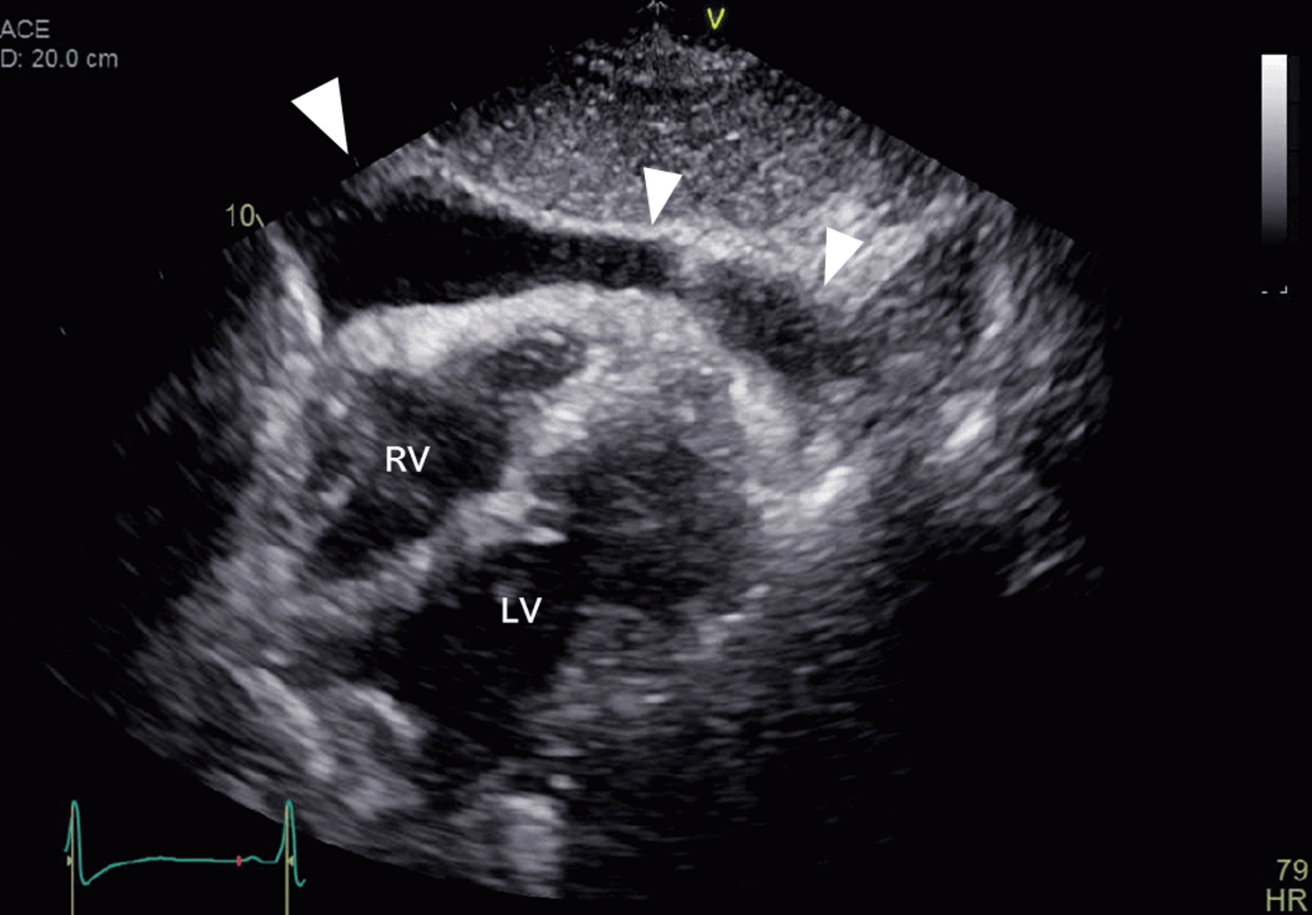
Echocardiographic image at the time of moderate pericardial effusion (in November 2024) On echocardiography, a moderate amount of pericardial effusion was predominantly located anterior to the right heart (white arrowhead), and right heart collapse was suspected RV: right ventricular; LV: left ventricular

In February of 2022, one month after treatment initiation, selpercatinib was discontinued due to hepatic dysfunction (common terminology criteria for adverse events ver. 5.0, grade 3). After improvement in liver function, treatment was resumed at a reduced dose of 160 mg/day (two-step dose reduction) in accordance with the package insert [12] and proper use guidelines [13]. In June 2023, a small amount of pericardial effusion was detected on chest CT, and by November 2024, further accumulation was observed. The patient’s vital signs were within normal limits, with a blood pressure of 132/70 mmHg and a regular heart rate of 80 beats/minute. No jugular venous distension or lower extremity edema was observed. On echocardiography (Figure 3), a moderate amount of pericardial effusion was predominantly located anterior to the right heart, and right heart collapse was suspected.

**Figure 4:**
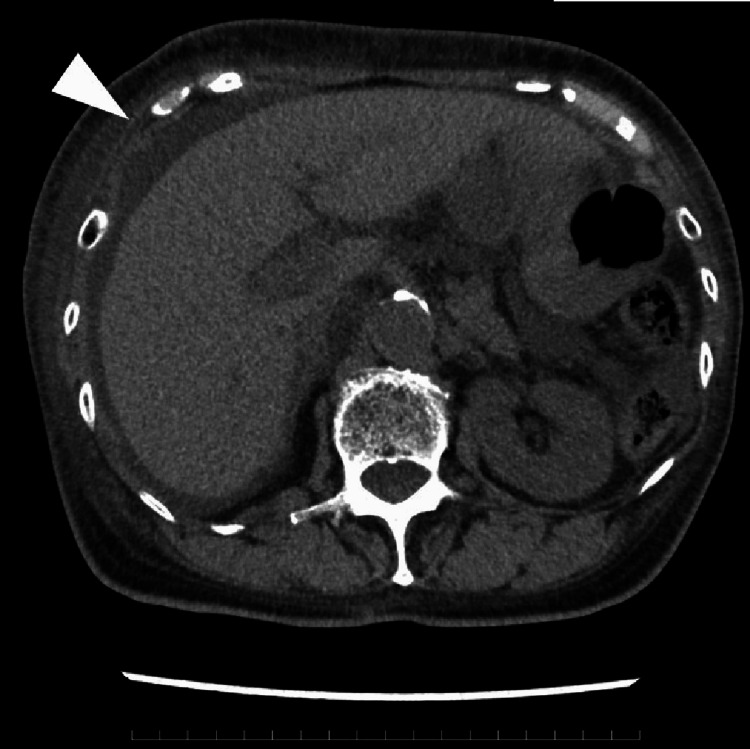
Ascites on abdominal computed tomography (in November 2024) A small amount of ascites (white arrow) were seen on the liver surface with increased pericardial fluid

However, no respiratory variation in ventricular inflow was observed, the inferior vena cava was not dilated, and normal respiratory variation was preserved. Therefore, the hemodynamic impact was considered limited. Pericardiocentesis could not be performed due to insufficient pericardial space for safe puncture under echocardiographic guidance. Additionally, the patient developed a complete left bundle branch block, accompanied by QT interval prolongation on electrocardiogram (ECG), along with the appearance of ascites (Figure 4).

**Figure 5:**
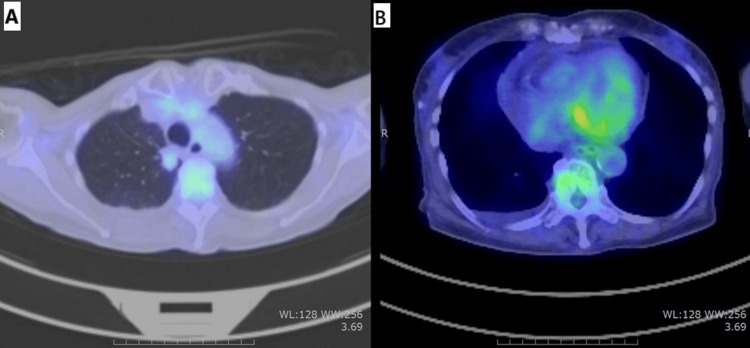
18F-FDG-PET/CT image at the time of pericardial effusion (on November 2024) The primary lesion and distant metastatic lesion also maintained a shrinking trend, and FDG accumulation was poor, so it was judged that there was no viable lung cancer lesion. (A) Primary lesion in the left upper lobe. (B) Pericardial area ^18^F-FDG-PET/CT: ^18^F-2-fluoro-2-deoxy-D-glucose positron-emission tomography/computed tomography

^18^F-2-fluoro-2-deoxy-D-glucose positron-emission tomography/computed tomography (18F-FDG-PET/CT) showed that the primary lesion and distant metastases continued to show regression, with poor FDG uptake (Figure 5), indicating no evidence of recurrence in her lung cancer.

Furthermore, laboratory tests revealed borderline anemia and mild hypoalbuminemia; however, liver and kidney function, as well as thyroid function, were within normal ranges. There were no inflammatory findings, uremia, or elevated antinuclear antibodies suggestive of autoimmune pericardial effusion (Table 1).

**Table 1:** Patient's laboratory results at the time of pericardial fluid increase (November 2024) WBC: white blood cells; RBC: red blood cells; Hb: hemoglobin; Ht: hematocrit; Plt: platelet; TP: total protein; Alb: albumin; T-bil: total bilirubin; AST: aspartate aminotransferase; ALT: alanine aminotransferase; LDH: lactate dehydrogenase; ALP: alkaline phosphatase; γ-GTP: gamma-glutamyl transferase; CK: creatinine kinase; Glu: glucose; UA: uric acid; BUN: blood urea nitrogen; Cre: creatinine; CRP: C-reactive protein; ANA: antinuclear antibody; TSH: thyroid stimulating hormone; Free T4: free thyroxine; Free T3: free triiodothyronine; CEA: carcinoembryonic antigen

Parameter	Value	Unit	Normal range
Blood cell counts			
WBC	4,200	/μL	3,300-8,600
Eosinophils	0.9	%	0.2-7.3
Basophils	0.0	%	0.2-2.0
Lymphocytes	23.1	%	21.3-50.2
Monocytes	4.2	%	2.7-7.6
Neutrophils	71.8	%	38.3-71.1
RBC	377 × 10^4^	/μL	386-492 × 10^4^
Hb	10.9	g/dL	11.6-14.8
Ht	34.7	%	34.6-44.6
Plt	24.2 × 10^4^	/μL	15.8-34.8
Biochemistry			
TP	5.7	g/dL	6.7-8.3
Alb	3.2	g/dL	3.8-5.2
T-bil	0.6	mg/dL	0.4-1.5
AST	23	U/L	13-30
ALT	19	U/L	7-23
LDH	188	U/L	124-222
ALP	70	U/L	38-113
γ-GTP	12	U/L	9-32
CK	122	U/L	45-163
Ca	8.5	mg/dL	8.5-10.2
UA	6.2	mg/dL	2.5-7.0
Glu	119	mg/dL	70-109
BUN	17	mg/dL	8.0-20.0
Cre	0.9	mg/dL	0.46-0.79
Na	144	mmol/L	138-145
K	4.6	mmol/L	3.6-4.8
Cl	102	mmol/L	101-108
Mg	2.1	mg/dL	1.8-2.6
Serology			
CRP	0.03	mg/dL	<0.14
ANA	<1:40	-	<1:40
Thyroid function			
TSH	3.47	μIU/mL	0.61-4.23
Free T3	1.95	pg/mL	2.1-3.8
Free T4	1.35	ng/dL	0.8-1.5
Tumor marker			
CEA	4.2	ng/mL	<5.0

Her echocardiography showed normal cardiac function, and there was no evidence of myocarditis, pericarditis, or other contributing medications other than selpercatinib. Since both the primary tumor and metastases had decreased in size following selpercatinib administration and no viable lesions were observed on ^18^F-FDG-PET/CT, we ruled out malignant pericardial effusion. In addition, the patient had no history of thoracic radiation therapy, antinuclear antibody testing was negative, and there were no accompanying inflammatory findings, making radiation-induced or autoimmune pericardial effusion unlikely. Although a definitive diagnosis of these conditions would have required analysis of the pericardial fluid via pericardiocentesis, echocardiography revealed insufficient space to safely perform the procedure. After consulting with a cardiologist, a small amount of pericardial effusion was detected approximately one year after selpercatinib administration was started, and since this pericardial effusion gradually increased thereafter, cardiotoxicity due to drug accumulation was suspected as the cause of the pericardial effusion.

Although no reports of pericardial effusion have been identified in the package insert, clinical trials, or existing case reports [9-13], we concluded that the pericardial effusion in this case was induced by selpercatinib based on the clinical course. Since there was a moderate amount of pericardial effusion, but not enough space to puncture and not enough to produce clinical symptoms, the patient was carefully followed for pericardial effusion. After the ECG improved, selpercatinib was restarted at a reduced dose of 80 mg/day (three-step reduction) but was withdrawn again due to the occurrence of left bundle branch block.

**Figure 6:**
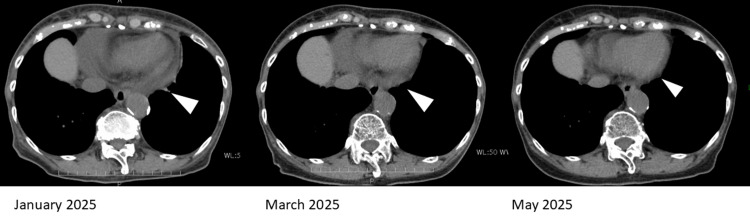
Pericardial effusion on CT (in January 2025: selpercatinib 80 mg/day; March and May 2025: selpercatinib 40 mg/day) Pericardial effusion observed in January 2025 while taking selpercatinib 80 mg/day gradually decreased after the selpercatinib dose was reduced to 40 mg/day (white arrow) CT: computed tomography

One month after discontinuation of selpercatinib, there were no further dose reduction options available for the adverse events, and because an increase in pericardial effusion could be life-threatening, switching to conventional chemotherapy was recommended. However, compared to the side effects experienced during first-line treatment with CBDCA+PEM, the patient reported milder symptoms from selpercatinib and expressed a desire to continue outpatient oral therapy and maintain her current quality of life. After a thorough discussion, selpercatinib was resumed at a further reduced dose of 40 mg/day. After resuming selpercatinib, careful follow-up with echocardiographic and electrocardiographic evaluations was conducted under the support of a cardiologist. One month after resuming selpercatinib, the complete left bundle branch block and pericardial effusion had decreased, and the tumor remained stable without progression (Figure 6).

Selpercatinib was continued at 40 mg/day for an additional two months. By May of year 2025, the pericardial fluid had further decreased; consequently, the same dose is still being administered.

Based on the clinical course and comprehensive diagnostic findings that excluded alternative causes of pericardial effusion, coupled with the observed reduction in effusion following selpercatinib dose reduction, we concluded that selpercatinib was the definitive cause of the pericardial effusion in this case.

## Discussion

To our knowledge, this is the first detailed case report suggesting the possibility of pericardial effusion as an adverse event of selpercatinib in a patient with lung cancer. Reports of pericardial effusion associated with selpercatinib have been included in the United States FDA Adverse Event Reporting System (FAERS) database from July 2020 to March 2023 [14]. However, the FAERS database lacks detailed clinical course information, and no detailed case reports describing selpercatinib-induced pericardial effusion have been published to date [9-13], leaving the clinical course of such adverse events unclear.

Selpercatinib exhibits high selectivity for RET; however, it also demonstrates weak inhibitory activity against vascular endothelial growth factor receptors (VEGFR) 1 and 3 in in vitro kinase assays [12]. VEGFR-3 is predominantly expressed in lymphatic endothelial cells [15], and its inhibition may be involved in known adverse events of selpercatinib, such as peripheral edema [16], chylothorax, and chylous ascites [17]. Furthermore, since VEGF plays a role in the regulation of pericardial fluid [18], selpercatinib may potentially induce pericardial effusion; however, accumulation of further case reports and studies is needed to clarify this association.

The median time to onset of peripheral edema caused by selpercatinib is reported to be 31.4 months [19], suggesting a tendency for delayed onset after prolonged treatment. Previous case reports have described small intestinal edema nine months after starting selpercatinib [20], as well as chylothorax and chylous ascites occurring more than six months after treatment initiation [20]. These findings highlight the importance of monitoring for fluid retention complications, including pericardial effusion, during long-term selpercatinib therapy. Especially when pericardial effusion develops gradually, distinguishing drug-induced effusion from malignant pericardial effusion due to progressive lung cancer is challenging. In this case, moderate pericardial effusion was observed; however, there was insufficient space on echocardiography to perform pericardiocentesis safely, and the characteristics of the pericardial fluid could not be confirmed. Other potential causes of pericardial effusion, such as cardiovascular disease, infection, autoimmune disorders, and radiation therapy, could only be excluded based on clinical course and other examinations, which is a limitation of this report.

Based on our experience, we recommend regular echocardiographic monitoring throughout the treatment period, not only during initial therapy but also in long-term follow-up. Cardiac monitoring should include both echocardiography and electrocardiography to detect early signs of cardiac toxicity, such as small amounts of pericardial effusion and conduction disturbances, before significant effusion develops. Regarding pericardial effusion, particular attention is needed during long-term observation, especially since delayed adverse effects may emerge 6-12 months after treatment initiation, as seen in this case.

In this patient, the selpercatinib dose was significantly reduced to 40 mg/day, which effectively alleviated adverse events, including pericardial effusion, and maintained tumor regression. Current adult treatment guidelines recommend discontinuation if adverse events persist despite dose reduction to 80 mg/day. However, after a thorough discussion with the patient regarding the risks and benefits, further dose reduction may be considered when patients prioritize quality of life and continued treatment, provided that close monitoring is maintained. As demonstrated in this case, keeping rare adverse events such as pericardial effusion in mind and conducting regular long-term monitoring may enable early detection of cardiac toxicity and allow dose adjustments that minimize side effects while maintaining therapeutic efficacy.

## Conclusions

This case provides a detailed discussion, including the clinical course, regarding the potential for selpercatinib-induced pericardial effusion. Although it is a single case report with inherent limitations, pericardial effusion may occur as an adverse event during long-term administration of selpercatinib, and clinicians should keep in mind that fluid retention-related side effects can develop over the course of prolonged treatment. Pericardial effusion can progress to severe and life-threatening conditions such as cardiac tamponade, and clinicians should be aware that such events may arise after extended treatment, as observed in this case. Therefore, thorough differential diagnosis should be performed whenever possible, and regular, long-term follow-up with echocardiography is necessary. Although no established criteria exist for dose reduction specifically for selpercatinib-induced pericardial effusion, dose reduction should be considered based on the severity of the adverse event, as it may lead to improvement.
